# Supplementary material for: Sialic acids in pancreatic cancer cells drive tumour-associated macrophage differentiation via the Siglec receptors Siglec-7 and Siglec-9
Source: Nat Commun. 2021 Feb 24;12:1270. doi: 10.1038/s41467-021-21550-4 (PMC7904912; doi:10.1038/s41467-021-21550-4)
Supplement: Supplementary file 1 — Supplementary Information [file 41467_2021_21550_MOESM1_ESM.pdf]

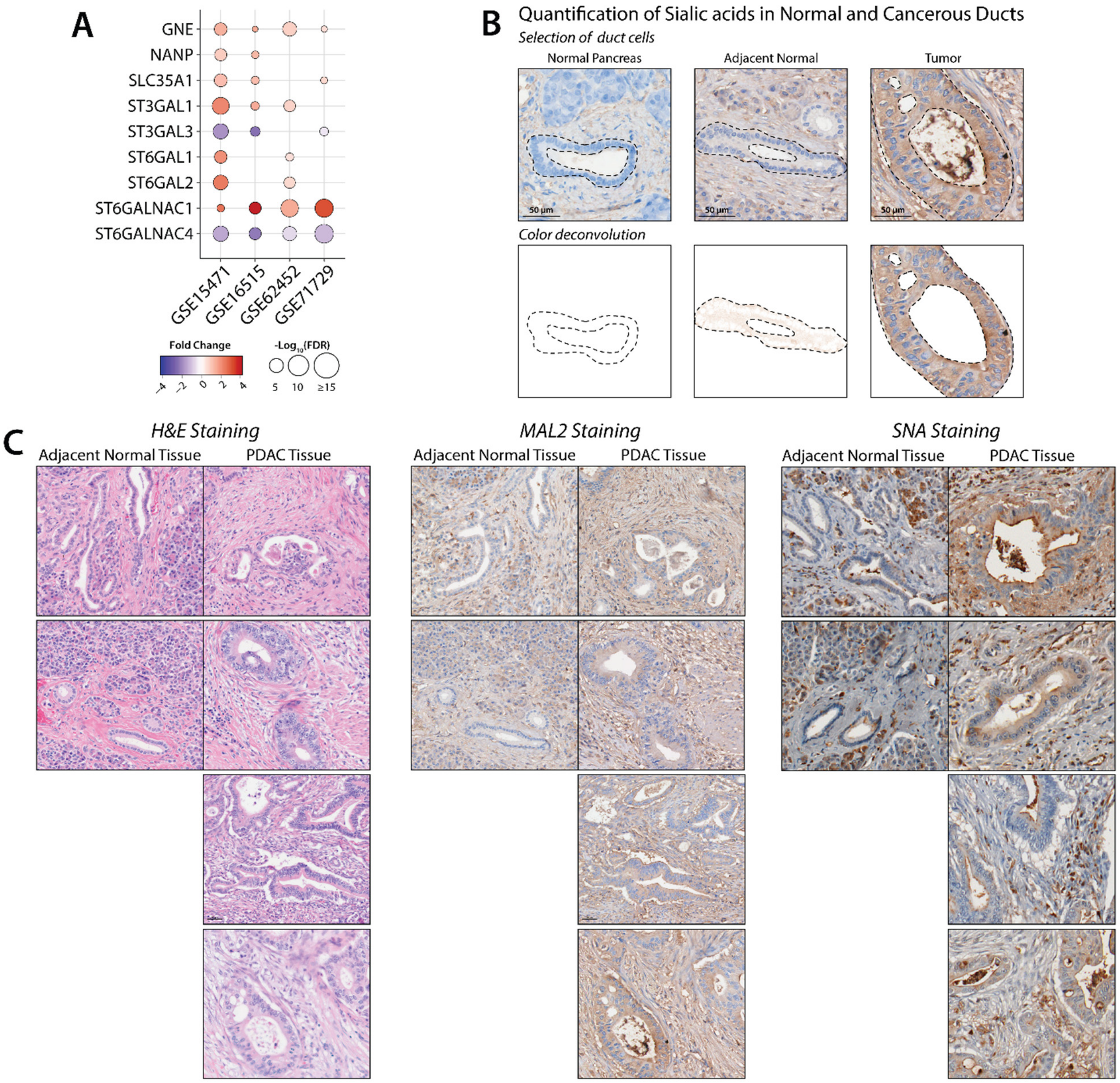

Supplementary Figure 1. PDAC present an increased expression of Sialic acid. Related to Figure 1. A) Differential expression of sialic acid related genes between tumor and normal tissue. B) Scheme representing the strategy for the quantification of sialic acid in ductal cells. Patients analyzed: MAL2 staining: adjacent normal (n = 5) and tumor (n = 8) tissue; SNA staining: adjacent normal (n = 8) and tumor (n = 8). C) Examples of hematoxilin and eosin (H&E) and immunohistochemistry staining in PDAC tissue and adjacent normal tissue.

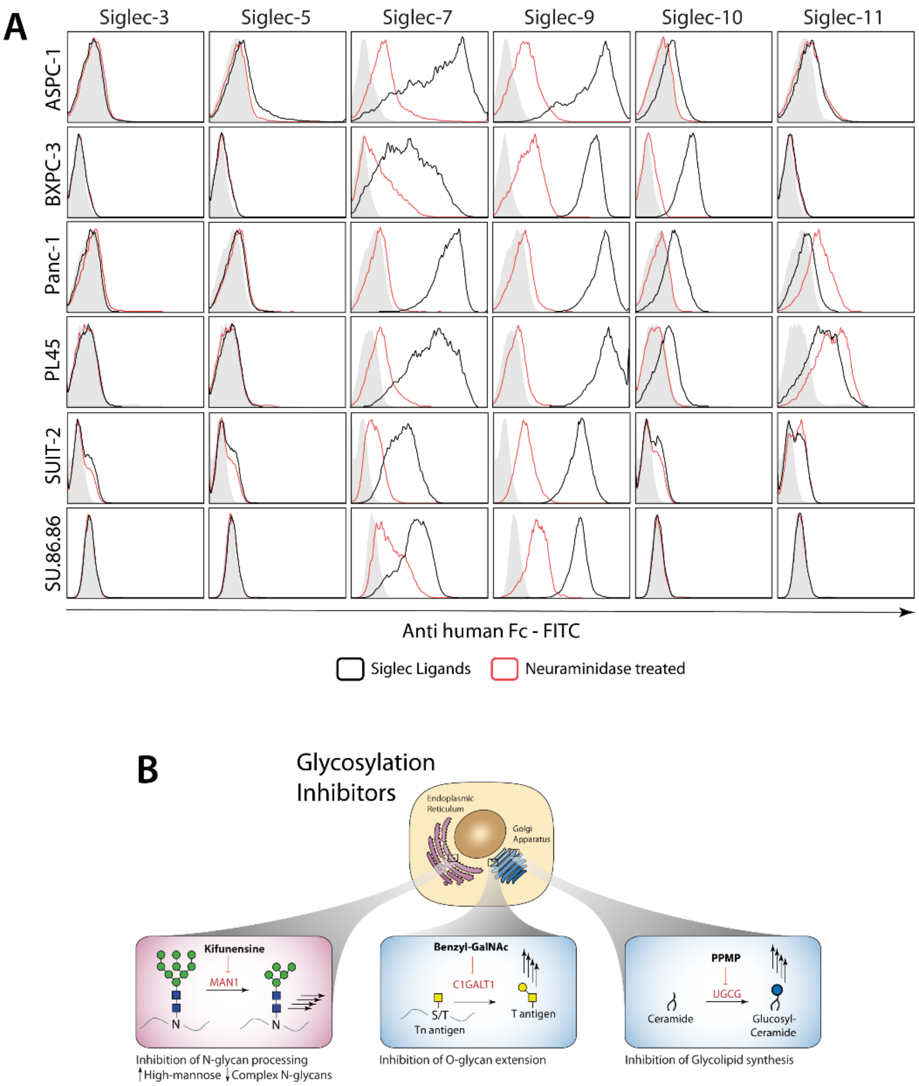

Supplementary Figure 2. PDAC cancer cells express ligands for Siglec-7 and Siglec-9. Related to Figure 2. A) Flow cytometry analysis of Siglecs ligand expression using Siglec-Fc chimeric constructs. B) Scheme representing the mechanisms of glycosylation inhibitors used in this study.

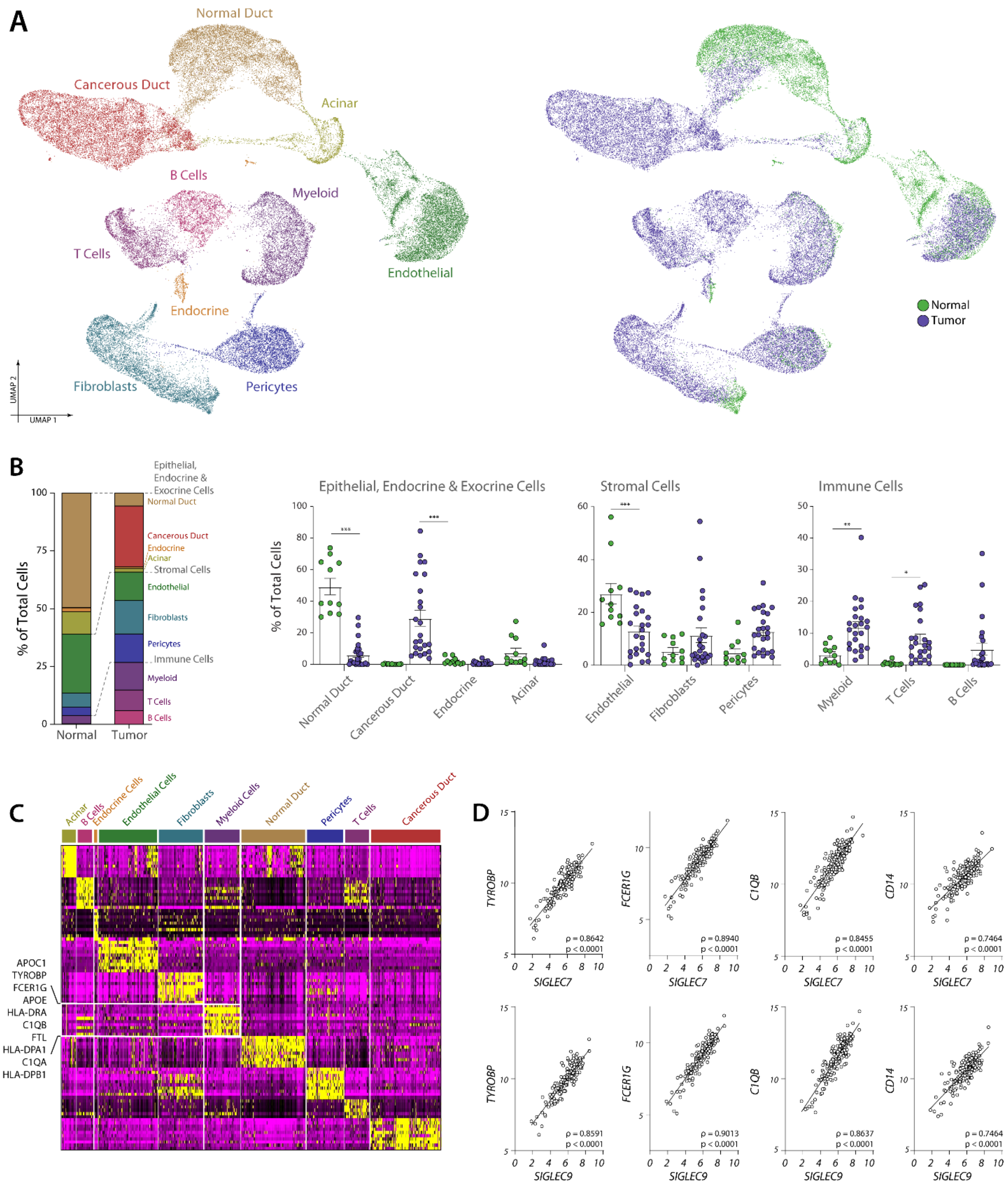

Supplementary Figure 3. Re-analysis of scRNA-seq previously published by *Peng et al*. Related to Figure 2 and 3. A) Identification of different cell populations in PDAC tissue. B) Quantification of different cell population in Normal (n = 11) and Tumor (n = 24) samples. Data presented as mean values ± SEM. Statistics: Two-way ANOVA with Dunnett's multiple comparisons test (* p ≤ 0.05; ** p ≤ 0.01; *** p ≤ 0.001). C) Heatmap of the different cell population identified in the scRNA-seq data, with some myeloid markers highlighted. D) Correlation of the gene expression of *SIGLEC7* and *SIGLEC9* with the one of myeloid markers *TYROBP*, *FCER1G*, *C1QB* and *CD14* in the TCGA cohort. Spearman correlation and p value is depicted in each graph.

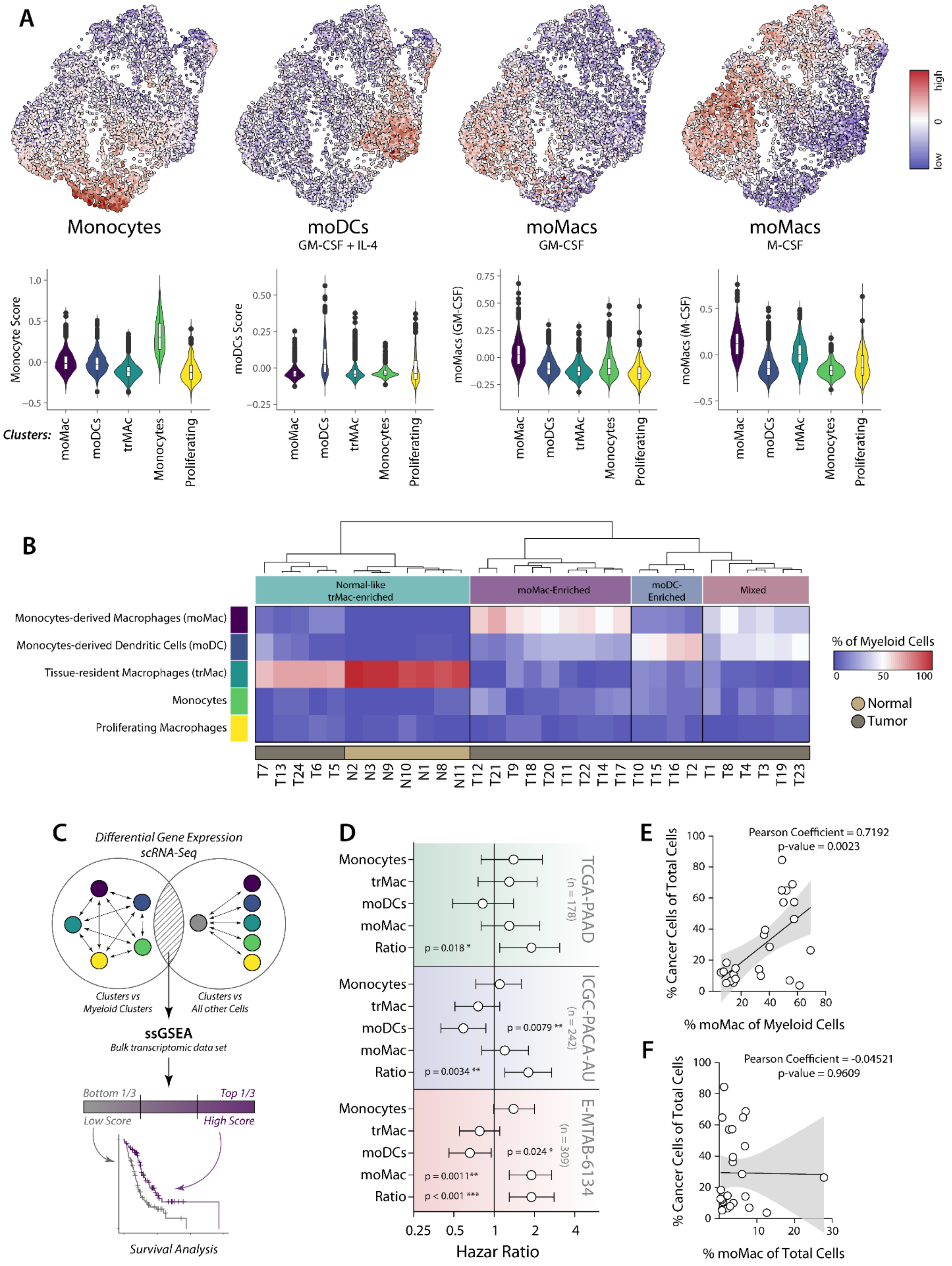

Supplementary Figure 4. Characterization of myeloid populations in PDAC. Related to Figure 3 and 4. A) Scores based on gene sets derived from *in vitro* induced moDC and moMac previously publish by Sander

et al [24]. Data presented as boxplot indicate the median, 25th and 75th percentiles (hinges) and whiskers represent 1.5 times the interquantile range. B) Heatmap representing the relative abundance of each myeloid subpopulation. C) Scheme representing the strategy for the generation of genesets specific for each myeloid population and its use in survival analysis. Top and bottom thirds scores selected to represent high and low scores, respectively. D) Univariate survival analysis in three different large datasets using the *Cox proportional hazards regression model*, as applied in the *coxph* function of the *survival* package in R. Total number of patients of each data sets are indicated in brackets. Data presented as Hazard ratio and 95% confidence interval. E-F) Correlation of the fraction of cancer cells in the samples with either the fraction of moMac in the Myeloid cells (E) or in the Total cells in the sample (F). Error bands correspond to 95% confidence interval.

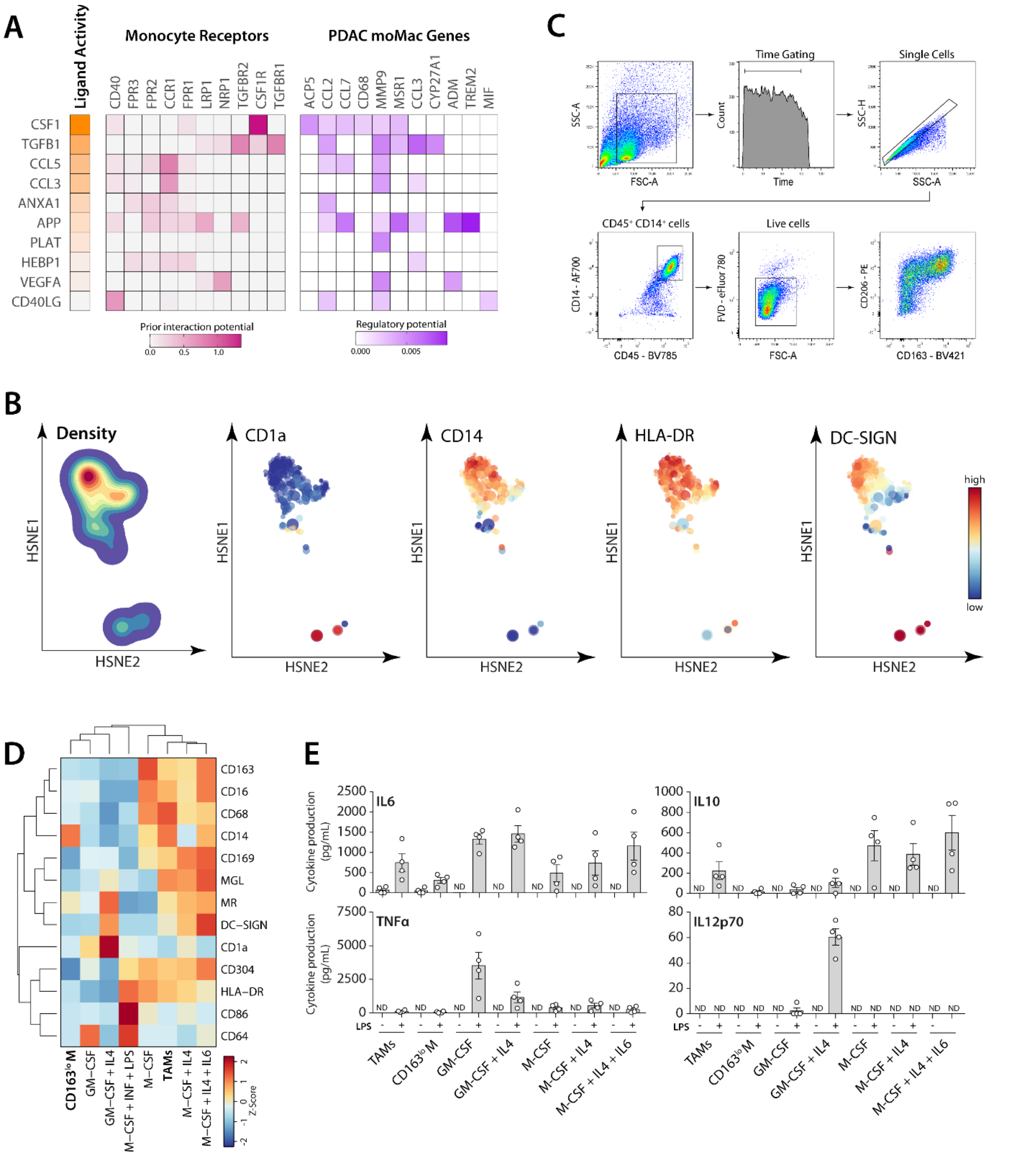

Supplementary Figure 5. Phenotype characterization after co-culture of monocytes with PDAC cell lines. Related to Figure 4. A) Niche net analysis of the cancer cell-derived factors and monocyte receptors that affect moMac differentiation. B) HSNE analysis of myeloid cells after co-culture. Display of density plot and expression of CD1a, CD14, HLA-DR and DC-SIGN. C) Gating strategy used for the analysis of marker expression after co-culture. D) Phenotyping of TAMs and CD163^{lo} myeloid cells compared to *in-vitro* induced dendritic cells and macrophage populations. Z-Score of FMO-corrected geometric mean of fluorescence intensity (ΔgMFI = MFI$_{Sample}$ - MFI$_{FMO}$). E) Cytokine production after LPS stimulation of sorted TAMs and CD163^{lo} myeloid cells or *in-vitro* differentiated dendritic cells and macrophage populations. Data presented as mean values ± SEM.

Supplementary Table 1. Characteristics of the datasets used in this paper.

**Differential gene expression**<br>Normal vs Tumor Tissue			
GSE ID	*n **	*Platform*	*Reference*
GSE15471	36 T / 36 NA	Microarray - Affymetrix Human Genome U133 Plus 2.0 Array	Badea, L. *et al* (2008) [1]
GSE16515	36 T / 16 N	Microarray - Affymetrix Human Genome U133 Plus 2.0 Array	Pei, H. *et al* (2009) [2]
GSE62452	69 T / 61 N	Microarray - Affymetrix Human Gene 1.0 ST Array	Shen, J. *et al* (2015) [3]
GSE71729	145 T / 49 N	Microarray - Agilent - 014850 Whole Human Genome Microarray 4x44K G4112F	Moffitt, R.A. *et al* (2015) [4]

**Single Cell RNA-Seq**<br>For characterization of the myeloid compartment in PDAC			
Study	*n**	*Platform*	*Reference*
PRJCA001063	24 T / 11 N	Illumina HiSeqXTen	Peng, *et al* (2019) [5]

**Large bulk transcriptomics data sets**<br>For Survival Analysis			
Study	*n***	*Platform*	*Reference*
TCGA PAAD	178	RNA-Seq - Illumina HiSeq 2000	Raphael, *et al* (2017) [6]
E-MTAB-6134	309	Microarray - Affymetrix Human Genome U219 Array	Puleo, F. *et al* (2018) [7]
ICGC – PACA – AU	242	Microarray - Illumina HumanHT-12 v4 Expression BeadChips	Bailey, P. *et al* (2016) [8]

* N: Normal Tissue; T: Tumor Tissue; NA: Normal Adjacent. ** All Tumor samples.

Supplementary Table 2. List of antibodies used in this paper. (C) Cytometry; (M) Microscopy.

Antibody	Dilution	Source	Catalogue No.
Flow Cytometry and Microscopy			
Anti-Siglec9 - AlexaFluor 594	1/50 (C)<br>1/1000 (M)	R&D Systems	FAB1139T
Anti-Siglec7 - AlexaFluor 647	1.100	R&D Systems	FAB11381R
Anti-Siglec7 – PerCP vio 700	1/50	Miltenyi	130-100-979
Anti-CD14 - AlexaFluor700	1/50	Sony	2109110
Streptavidin - AlexaFluor 555	1/600	ThermoFisher Scientific	S32355
Anti-CD45 - Brilliant Violet 785	1/100	Biolegend	304031
Streptavidin - AlexaFluor 647	1/400	ThermoFisher Scientific	S32357
DAPI	1/1000	Invitrogen	J11372
Anti-PanCytokeratine - AlexaFluor 488	1/100	eBioscience	53-9003-82
Anti-CD206 - PE	1/50	Biolegend	321106
Anti-CD163 - Brilliant Violet 421™	1/50	Biolegend	333612
Anti-CD86 - Brilliant Violet 650™	1/75	Biolegend	305427
Anti-DC-SIGN (AZN-D1) - AlexaFluor 488	1/50	In house	
Anti-HLA-DR - BV786	1/100	BD Biosciences	564041
Anti-CD1a - APC	1/50	Biolegend	300110
Anti-PD-L1 - Pe-Cy7	1/200	Biolegend	329718
Anti-MGL (CD301) - PE	1/100	Biolegend	354703
Anti-CD68 - AlexaFluor 488	1/100	Biolegend	333811
Anti-CD169 - AlexaFluor 647	1/50	Novus	NB600-534AF647
Anti-CD64 - Pe-Cy7	1/100	Biolegend	305022
Anti-CD304 - APC-R700	1/100	BD Biosciences	566039
Anti-human IgG - FITC	1/50	Jackson ImmunoResearch	109-096-098
Blocking experiments			
Purified anti-human Siglec-7		Biolegend	347702
Purified anti-human Siglec-9		R&D Systems	MAB1139-100
ELISA			
Capture Antibody - IL-10	1/2000	eBioscience	14-7108-85
Detection Antibody - IL-10	1/2000	eBioscience	13-7109-85
Capture Antibody - IL-12p40	1/1000	eBioscience	14-7128-82
Detection Antibody - IL-12(p40/p70)	1/1000	eBioscience	13-7129-81
Capture Antibody - IL-6	1/2000	Biosource	AHC0562
Detection Antibody - IL-6	1/2500	Biosource	AHC0469
Capture Antibody - TNFα	1/1000	Biosource	AHC3712
Detection Antibody - TNFα	1/1000	Biosource	AHC3419
Capture Antibody – IL1β	1/1000	Biosource	AHC0612
Detection Antibody - IL1β	1/700	Biosource	AHC0519

Supplementary References

1. Badea L, Herlea V, Dima SO, Dumitrascu T and Popescu I, *Combined gene expression analysis of whole-tissue and microdissected pancreatic ductal adenocarcinoma identifies genes specifically overexpressed in tumor epithelia.* Hepatogastroenterology, 2008. **55**(88): p. 2016-27.
2. Pei H, Li L, Fridley BL, Jenkins GD, Kalari KR, Lingle W, et al., *FKBP51 affects cancer cell response to chemotherapy by negatively regulating Akt.* Cancer Cell, 2009. **16**(3): p. 259-66.
3. Shen J, Xiao Z, Wu WK, Wang MH, To KF, Chen Y, et al., *Epigenetic silencing of miR-490-3p reactivates the chromatin remodeler SMARCD1 to promote Helicobacter pylori-induced gastric carcinogenesis.* Cancer Res, 2015. **75**(4): p. 754-65.
4. Moffitt RA, Marayati R, Flate EL, Volmar KE, Loeza SG, Hoadley KA, et al., *Virtual microdissection identifies distinct tumor- and stroma-specific subtypes of pancreatic ductal adenocarcinoma.* Nat Genet, 2015. **47**(10): p. 1168-78.
5. Peng J, Sun BF, Chen CY, Zhou JY, Chen YS, Chen H, et al., *Single-cell RNA-seq highlights intra-tumoral heterogeneity and malignant progression in pancreatic ductal adenocarcinoma.* Cell Res, 2019.
6. Raphael BJ, Hruban RH, Aguirre AJ, Moffitt RA, Yeh JJ, Stewart C, et al., *Integrated Genomic Characterization of Pancreatic Ductal Adenocarcinoma.* Cancer Cell, 2017. **32**(2): p. 185-203.e13.
7. Puleo F, Nicolle R, Blum Y, Cros J, Marisa L, Demetter P, et al., *Stratification of Pancreatic Ductal Adenocarcinomas Based on Tumor and Microenvironment Features.* Gastroenterology, 2018. **155**(6): p. 1999-2013 e3.
8. Bailey P, Chang DK, Nones K, Johns AL, Patch AM, Gingras MC, et al., *Genomic analyses identify molecular subtypes of pancreatic cancer.* Nature, 2016. **531**(7592): p. 47-52.
