# Supplementary material for: Sialic acids in pancreatic cancer cells drive tumour-associated macrophage differentiation via the Siglec receptors Siglec-7 and Siglec-9
Source: Nat Commun. 2021 Feb 24;12:1270. doi: 10.1038/s41467-021-21550-4 (PMC7904912; doi:10.1038/s41467-021-21550-4)
Supplement: Supplementary file 3 — Description of Additional Supplementary Files [file 41467_2021_21550_MOESM3_ESM.pdf]

Description of Additional Supplementary Files

File Name: Supplementary Data 1
Description: Differential gene expression between the identified myeloid cells in the scRNA-Seq from Peng et al.

File Name: Supplementary Data 2
Description: Gene Sets used for the characterization of myeloid cells based from Sander et al.
